# Supplementary material for: A portable solution for simultaneous human movement and mobile EEG acquisition: readiness potential for basketball free-throw shooting
Source: Exp Brain Res. 2026 Jul 7;244(8):153. doi: 10.1007/s00221-026-07342-6 (PMC13342168; doi:10.1007/s00221-026-07342-6)
Supplement: Supplementary file 1 — Supplementary Material 1 [file 221_2026_7342_MOESM1_ESM.zip › Supplementary Information/Supplementary Information.docx]

Supplementary Information (SI)

# Supplementary Figures


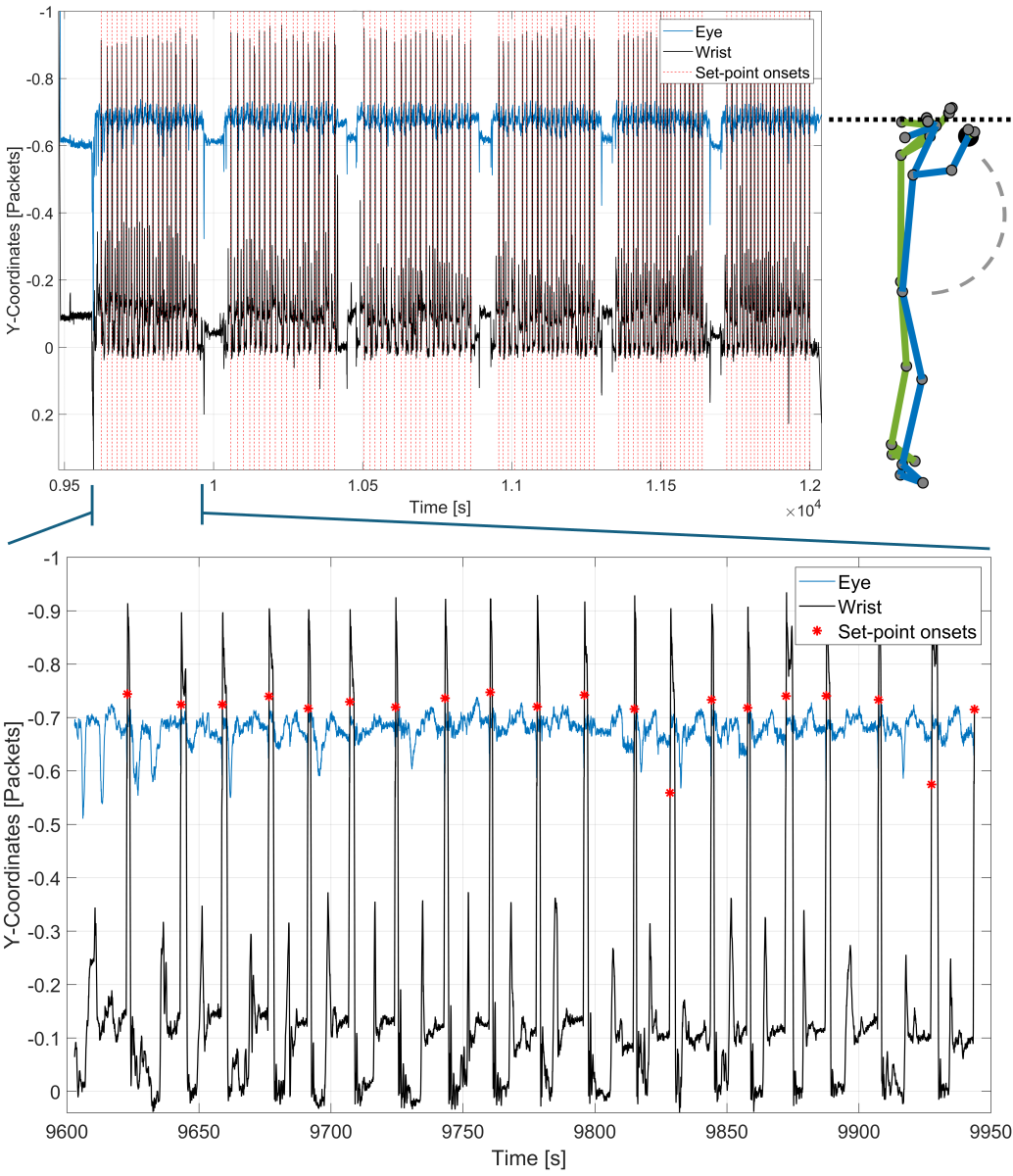


**SI Fig 1 Set-point onset detection for basketball shooting.** Pose Landmark Detection (PLD) data is used to determine a reference time for free throw attempts. This method selects time references based on Y-coordinates of body points. The start of the event is detected when the wrist of the dominant side of the participant is elevated above the eye level. This eye-wrist intersection represents the stereotypical movement for a basketball player called the “set-point”. The wrist (black line) and eye (blue line) intersections are plotted over time, showing the 20 shots x 6 blocks with the break rest period for a participant.

​​


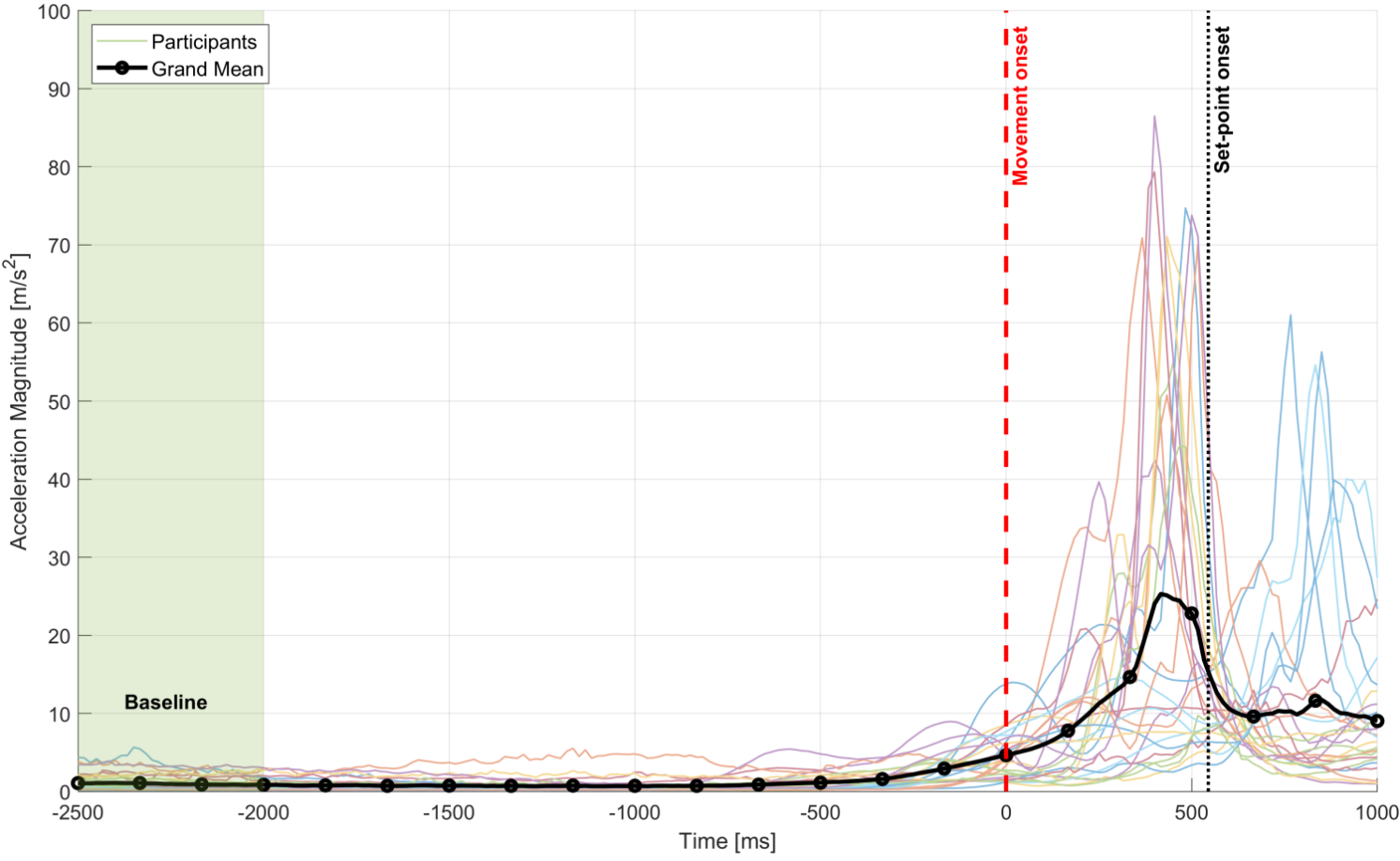


**SI Fig 2 Movement onset detection based on reverse computation algorithm.** The acceleration magnitude of wrist movement over time is calculated from the signals of the Movella DOT sensor attached to the right wrist of participants, with single lines in light colors representing each participant’s mean across trials. The grand average acceleration across participants in showed in black. The baseline period is highlighted in light green. The detected average movement onset time is indicated by the red dashed line and the average “set-point” onset time is indicated by the black dot line.


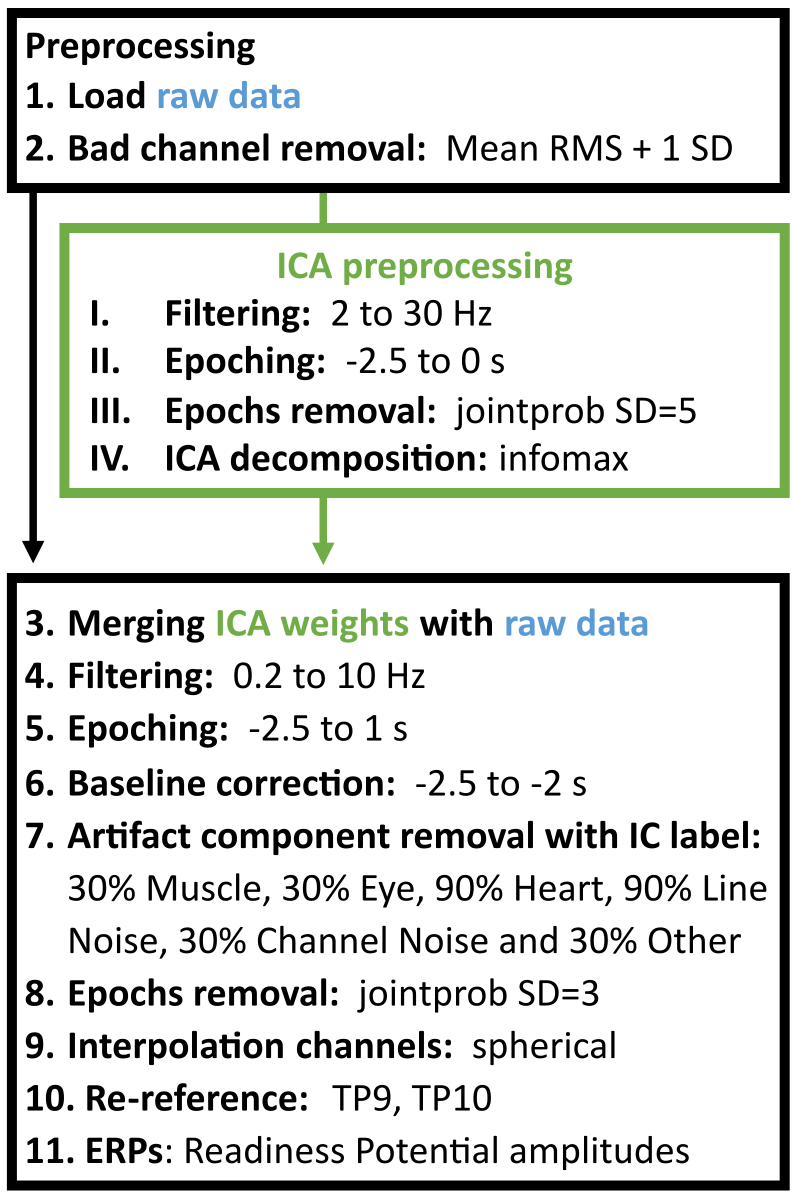


**SI Fig 3 EEG preprocessing pipeline.** Independent Component Analysis (ICA) was applied**.** To obtain the ICA weights, bad channels were rejected, data was filtered, bad epochs were removed, and an extended infomax ICA was run. The resulting ICA weights were applied to the unfiltered, continuous raw data for further preprocessing (merging ICA weights with the raw data). At the end the RP was parameterized and submitted to statistical analysis.


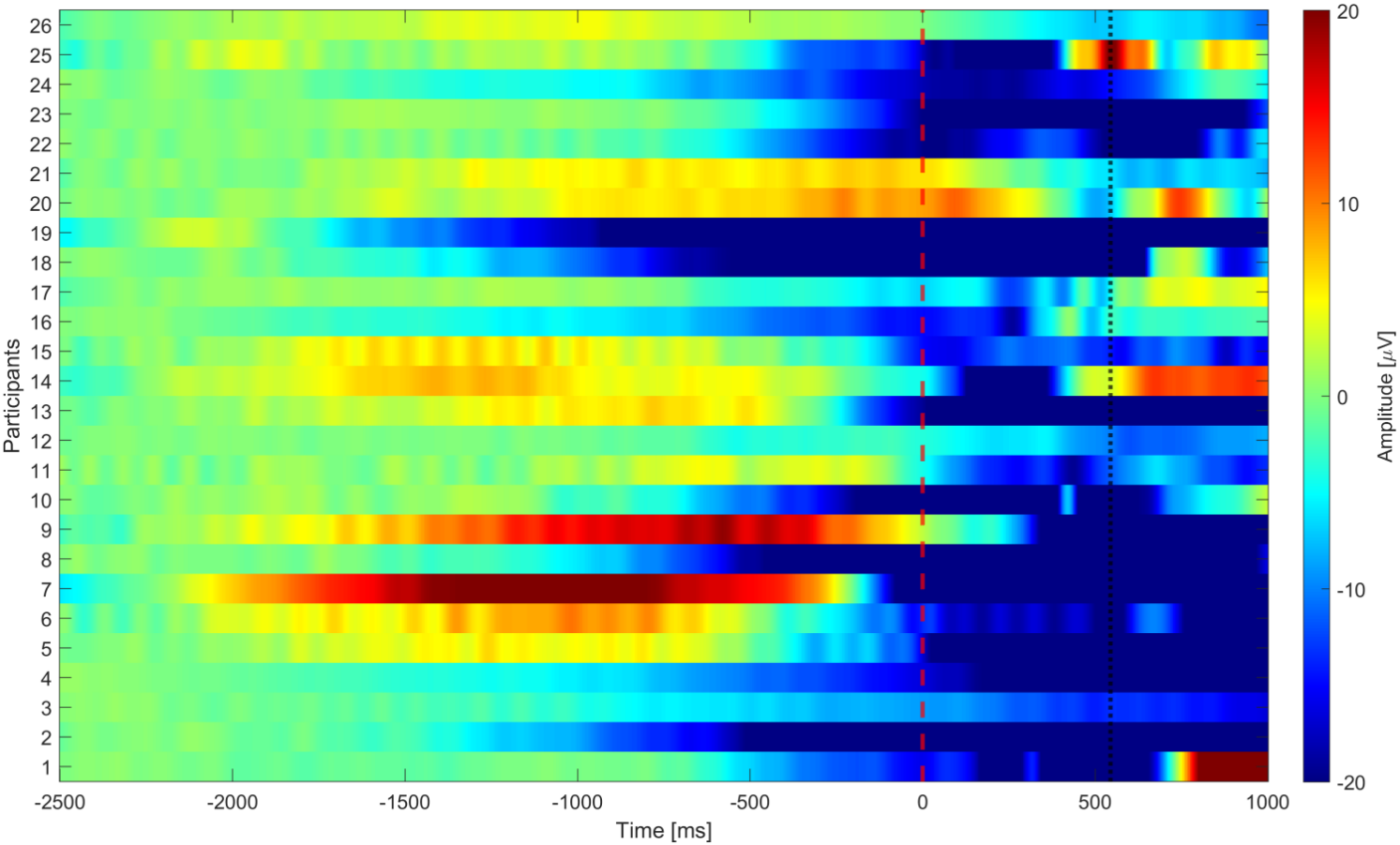


**SI Fig 4 Readiness potential of each participant at channel Cz.** The heatmap displays the RP at channel Cz for 26 participants, aligned to movement onset (time 0, marked by a red dashed line). Each row corresponds to a participant's ERP, with amplitudes represented by the color scale on the right. The time axis spans from -2500 ms to 1000 ms relative to movement onset. The vertical black dotted line indicates the average “set-point” onset.


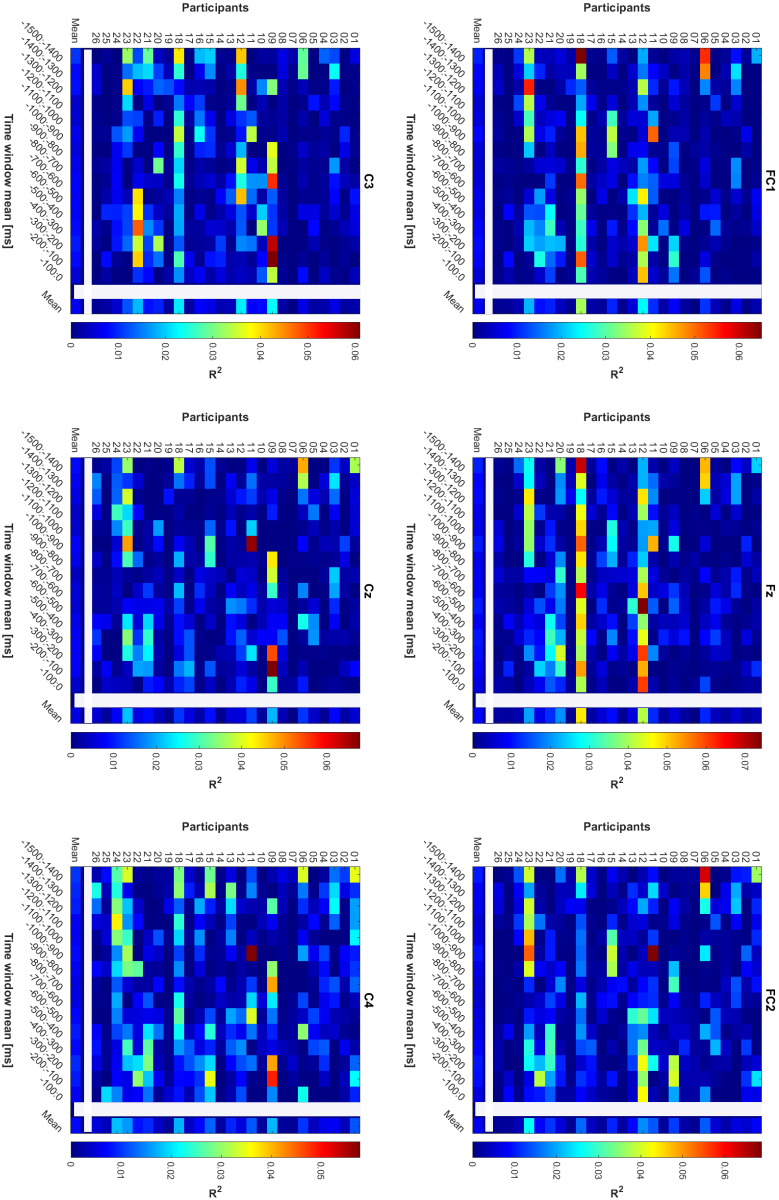


**SI Fig 5** E**xplained variance (*R*²) from point bi-serial correlation of single-trial amplitudes** **between hits and misses across participants.** Heat-maps represent the explained variance (*R*²) of RP features (15 time windows of 100 ms prior movement) at channels C3, Cz, C4, FC1, Fz, and FC2. Each heat-map shows the proportion of variance in trial outcomes explained by each feature across 26 participants. Red colors indicate higher *R*² values (more variance explained), while blue colors indicate lower *R*² values (less variance explained). The mean *R*² value across participants is shown as the last row in each heat-map as well as the mean across features as the last column, providing an overall indication of the most significant features for distinguishing between hits and misses.


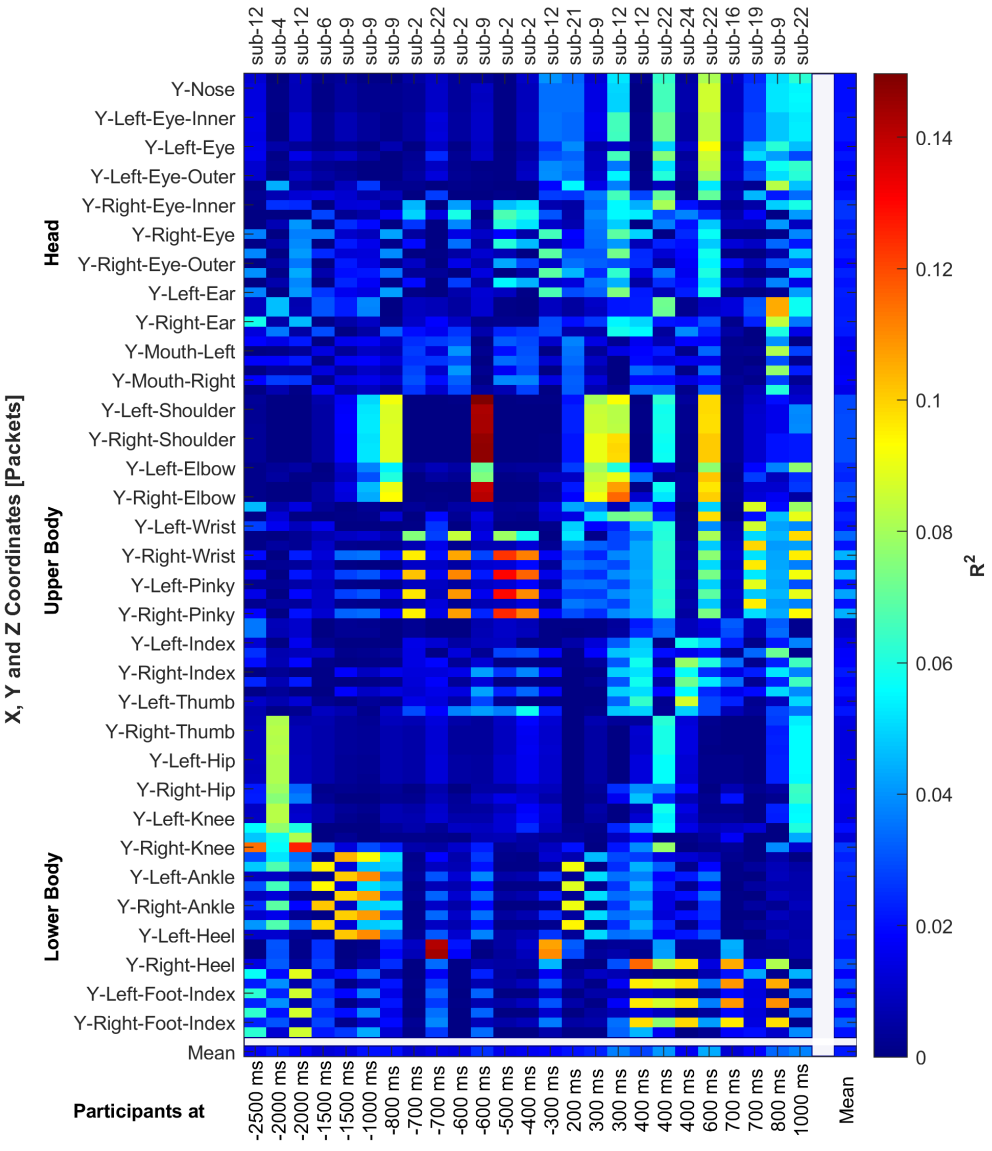


**SI Fig 6** E**xplained variance (*R*²) from point bi-serial correlation of the pose landmarks between hits and misses across participants.** The heatmap illustrates the explained variance (*R*²) derived from point-biserial correlation analysis, highlighting pose differences between hits and misses across participants over time. Each row represents a specific body landmark, categorized into “head”, “upper body”, and “lower body”, while each column corresponds to a participant or the mean (rightmost column). The color scale indicates the magnitude of *R*² values, with warmer colors reflecting greater explained variance. Time windows (100 ms bins) span from -2500 ms to 1000 ms relative to movement onset.

# Supplementary Tables

**SI Table 1. Specifications of the basketball profile for all participants.**

| **Specifications of the basketball profile for all participants** | | | | | | | |
| --- | --- | --- | --- | --- | --- | --- | --- |
| **Participant** | **Age** | **Basketball club membership (years)** | **Basketball experience (years)** | **Basketball practice (hours per week)** | **Last contact with basketball (weeks)** | **Basketball tournaments participation** | **Own performance estimation (%)** |
| **sub_01** | 30 | 3 | 15 | 3 | 1 | Yes | 80 |
| **sub_02** | 25 | 0 | 3 | 2 | 1 | No | 20 |
| **sub_03** | 22 | 3 | 3 | 3 | 1 | Yes | 65 |
| **sub_04** | 22 | 0 | 7 | 2 | 1 | Yes | 45 |
| **sub_05** | 28 | 5 | 4.5 | 3 | 1 | Yes | 30 |
| **sub_06** | 35 | 0 | 7 | 3 | 1 | Yes | 50 |
| **sub_07** | 26 | 3 | 6 | 4 | 1 | Yes | 30 |
| **sub_08** | 21 | 10 | 10 | 1 | 1 | Yes | 60 |
| **sub_09** | 19 | 4 | 3 | 2 | 1 | Yes | 60 |
| **sub_10** | 21 | 0 | 3 | 3 | 1 | Yes | 60 |
| **sub_11** | 24 | 3 | 3 | 1 | 1 | No | 35 |
| **sub_12** | 23 | 15 | 8 | 2 | 1 | Yes | 60 |
| **sub_13** | 21 | 7 | 7 | 2 | 1 | Yes | 70 |
| **sub_14** | 23 | 0 | 3 | 1 | 1 | Yes | 50 |
| **sub_15** | 18 | 9 | 8 | 4 | 1 | Yes | 60 |
| **sub_16** | 23 | 18 | 5 | 1 | 1 | No | 30 |
| **sub_17** | 24 | 10 | 7 | 4 | 1 | Yes | 90 |
| **sub_18** | 19 | 11 | 4 | 2 | 1 | Yes | 50 |
| **sub_19** | 22 | 16 | 10 | 2 | 1 | Yes | 70 |
| **sub_20** | 24 | 0 | 3 | 1 | 1 | Yes | 60 |
| **sub_21** | 30 | 0 | 12 | 1 | 1 | Yes | 60 |
| **sub_22** | 25 | 0 | 11 | 4 | 1 | No | 80 |
| **sub_23** | 23 | 3 | 4 | 4 | 1 | Yes | 50 |
| **sub_24** | 29 | 0 | 9 | 5 | 1 | Yes | 50 |
| **sub_25** | 32 | 18 | 16 | 2 | 1 | Yes | 80 |
| **sub_26** | 31 | 0 | 5 | 2 | 1 | No | 70 |

**SI Table 2. Results of the data lost during EEG preprocessing and the measurement of basketball shooting accuracy for all participants.**

| **Participants specifications (EEG preprocessing & free-throw accuracy)** | | | | | | | |
| --- | --- | --- | --- | --- | --- | --- | --- |
| **Participant** | **Hits** | **Misses** | **Accuracy (%)** | **EEG**  **Rejected components** | **EEG Rejected Trials** | **EEG good trials** | **EEG**  **rejected channels** |
| **sub_01** | 79 | 26 | 75.2 | 20 | 15 | 105 | 4 |
| **sub_02** | 34 | 70 | 32.6 | 13 | 16 | 104 | 5 |
| **sub_03** | 69 | 35 | 66.3 | 19 | 16 | 104 | 2 |
| **sub_04** | 30 | 73 | 29.1 | 15 | 17 | 103 | 6 |
| **sub_05** | 52 | 49 | 51.4 | 3 | 19 | 101 | 5 |
| **sub_06** | 36 | 64 | 36.0 | 6 | 20 | 100 | 4 |
| **sub_07** | 27 | 83 | 24.5 | 12 | 10 | 110 | 5 |
| **sub_08** | 20 | 90 | 18.1 | 13 | 10 | 110 | 6 |
| **sub_09** | 62 | 35 | 63.9 | 10 | 23 | 97 | 4 |
| **sub_10** | 43 | 62 | 40.9 | 10 | 15 | 105 | 5 |
| **sub_11** | 28 | 74 | 27.4 | 8 | 18 | 102 | 1 |
| **sub_12** | 24 | 81 | 22.8 | 15 | 15 | 105 | 6 |
| **sub_13** | 74 | 29 | 71.8 | 17 | 17 | 103 | 5 |
| **sub_14** | 24 | 78 | 23.5 | 16 | 18 | 102 | 5 |
| **sub_15** | 83 | 30 | 73.4 | 11 | 7 | 113 | 5 |
| **sub_16** | 54 | 48 | 52.9 | 15 | 18 | 102 | 5 |
| **sub_17** | 86 | 18 | 82.6 | 15 | 16 | 104 | 4 |
| **sub_18** | 42 | 62 | 40.3 | 19 | 16 | 104 | 4 |
| **sub_19** | 70 | 38 | 64.8 | 10 | 12 | 108 | 6 |
| **sub_20** | 67 | 35 | 65.6 | 18 | 18 | 102 | 1 |
| **sub_21** | 61 | 45 | 57.5 | 10 | 14 | 106 | 6 |
| **sub_22** | 68 | 29 | 70.1 | 10 | 23 | 97 | 5 |
| **sub_23** | 58 | 46 | 55.7 | 11 | 16 | 104 | 6 |
| **sub_24** | 64 | 38 | 62.7 | 19 | 18 | 102 | 3 |
| **sub_25** | 78 | 23 | 77.2 | 12 | 19 | 101 | 3 |
| **sub_26** | 69 | 37 | 65.0 | 15 | 14 | 106 | 2 |

**SI Table 3. Results of Wilcoxon signed-rank test for a single mean ERP amplitude against zero for each participant.** To evaluate the presence of the RP at individual-level, the mean ERP amplitude across time at channel Cz of each participant was tested (from -1500 to 0 ms). The table includes the following metrics for each participant: mean ERP amplitude (*μV*), standard error (SE), z-score (*z*), signed rank, degrees of freedom (*df*), p-value (*p*), and effect size (*d*). Significance levels are indicated as follows: *p < 0.05***, p < 0.0******,*** *p < 0.001****. Negative mean amplitudes reflect the presence of a RP, with its corresponding significance, suggesting that the ERP deviates from baseline in anticipation of movement.

| **Wilcoxon signed-rank test - Single mean ERP vs 0 at channel Cz** | | | | | | | |
| --- | --- | --- | --- | --- | --- | --- | --- |
| **Participant** | **Mean (*μV*)** | **SE (*μV*)** | ***z*** | **Signed-rank** | ***df*** | ***p*** | ***d*** |
| **sub_01** | -4.68 | 0.72 | -5.49 | 1066 | 104 | 8.18E-08*** | -0.63 |
| **sub_02** | -15.88 | 1.46 | -7.93 | 286 | 103 | 9.85E-15*** | -1.07 |
| **sub_03** | -5.21 | 0.70 | -6.24 | 806 | 103 | 1.27E-09*** | -0.73 |
| **sub_04** | -8.42 | 0.73 | -8.03 | 238 | 102 | 5.18E-15*** | -1.14 |
| **sub_05** | 0.46 | 1.03 | -0.30 | 2488 | 100 | 0.76691073 | 0.04 |
| **sub_06** | 2.69 | 0.99 | 2.57 | 3273 | 99 | 0.013841819** | 0.27 |
| **sub_07** | 12.96 | 4.34 | 3.20 | 4126 | 109 | 0.002221594** | 0.28 |
| **sub_08** | -12.96 | 3.19 | -3.60 | 1846 | 109 | 0.000555519*** | -0.39 |
| **sub_09** | 13.22 | 0.82 | 8.36 | 4701 | 96 | 3.94E-16*** | 1.64 |
| **sub_10** | -7.44 | 0.93 | -6.57 | 726 | 104 | 1.59E-10*** | -0.78 |
| **sub_11** | 1.74 | 0.58 | 2.82 | 3471 | 101 | 0.007365543** | 0.30 |
| **sub_12** | -1.79 | 0.77 | -2.19 | 2099 | 104 | 0.037553634* | -0.23 |
| **sub_13** | 1.41 | 0.83 | 1.02 | 2987 | 102 | 0.32172501 | 0.17 |
| **sub_14** | 3.93 | 1.61 | 1.91 | 3199 | 101 | 0.06617067 | 0.24 |
| **sub_15** | 1.35 | 0.94 | 1.12 | 3611 | 112 | 0.28519705 | 0.14 |
| **sub_16** | -7.96 | 0.51 | -8.48 | 87 | 101 | 2.22E-16*** | -1.53 |
| **sub_17** | -1.43 | 0.85 | -2.08 | 2090 | 103 | 0.046985473* | -0.16 |
| **sub_18** | -17.11 | 0.89 | -8.74 | 34 | 103 | 5.92E-17*** | -1.89 |
| **sub_19** | -22.99 | 2.20 | -7.58 | 470 | 107 | 1.28E-13*** | -1.01 |
| **sub_20** | 5.51 | 0.75 | 6.05 | 4438 | 101 | 3.83E-09*** | 0.72 |
| **sub_21** | 5.20 | 1.47 | 3.76 | 4030 | 105 | 0.000309389*** | 0.34 |
| **sub_22** | -5.79 | 0.37 | -8.46 | 24 | 96 | 2.22E-16*** | -1.61 |
| **sub_23** | -3.48 | 0.54 | -5.49 | 1038 | 103 | 8.18E-08*** | -0.63 |
| **sub_24** | -8.13 | 1.12 | -6.00 | 829 | 101 | 4.65E-09*** | -0.72 |
| **sub_25** | -3.12 | 1.11 | -2.80 | 1749 | 100 | 0.007384192** | -0.28 |
| **sub_26** | 2.65 | 1.33 | 1.76 | 3393 | 105 | 0.08917705 | 0.19 |
