# Supplementary figures and images for: A portable solution for simultaneous human movement and mobile EEG acquisition: readiness potential for basketball free-throw shooting
Source: Exp Brain Res. 2026 Jul 7;244(8):153. doi: 10.1007/s00221-026-07342-6 (PMC13342168; doi:10.1007/s00221-026-07342-6)

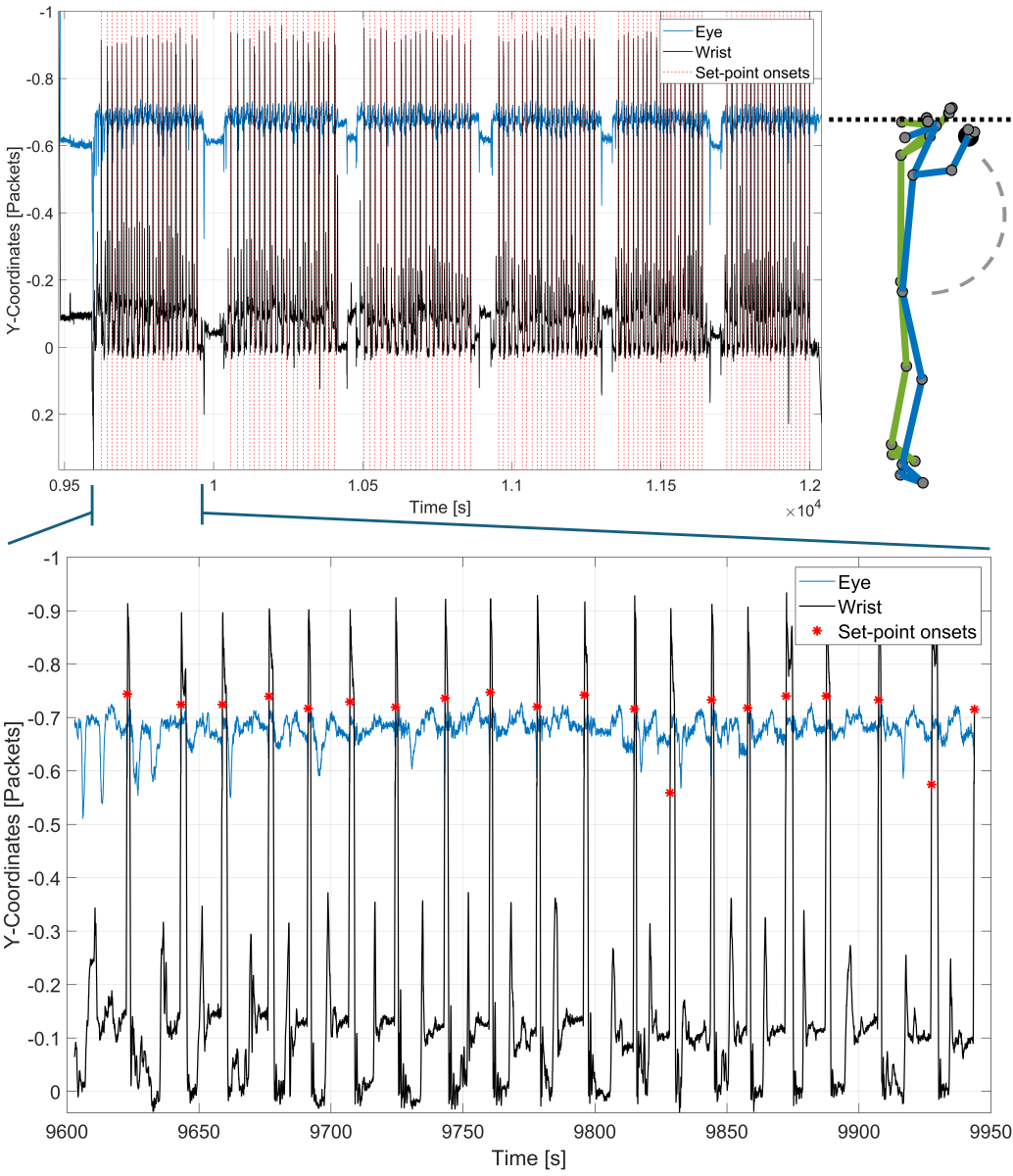

Supplement: Supplementary file 1 — Supplementary Material 1 [file 221_2026_7342_MOESM1_ESM.zip › Supplementary Information/Supplementary_Fig1.png]

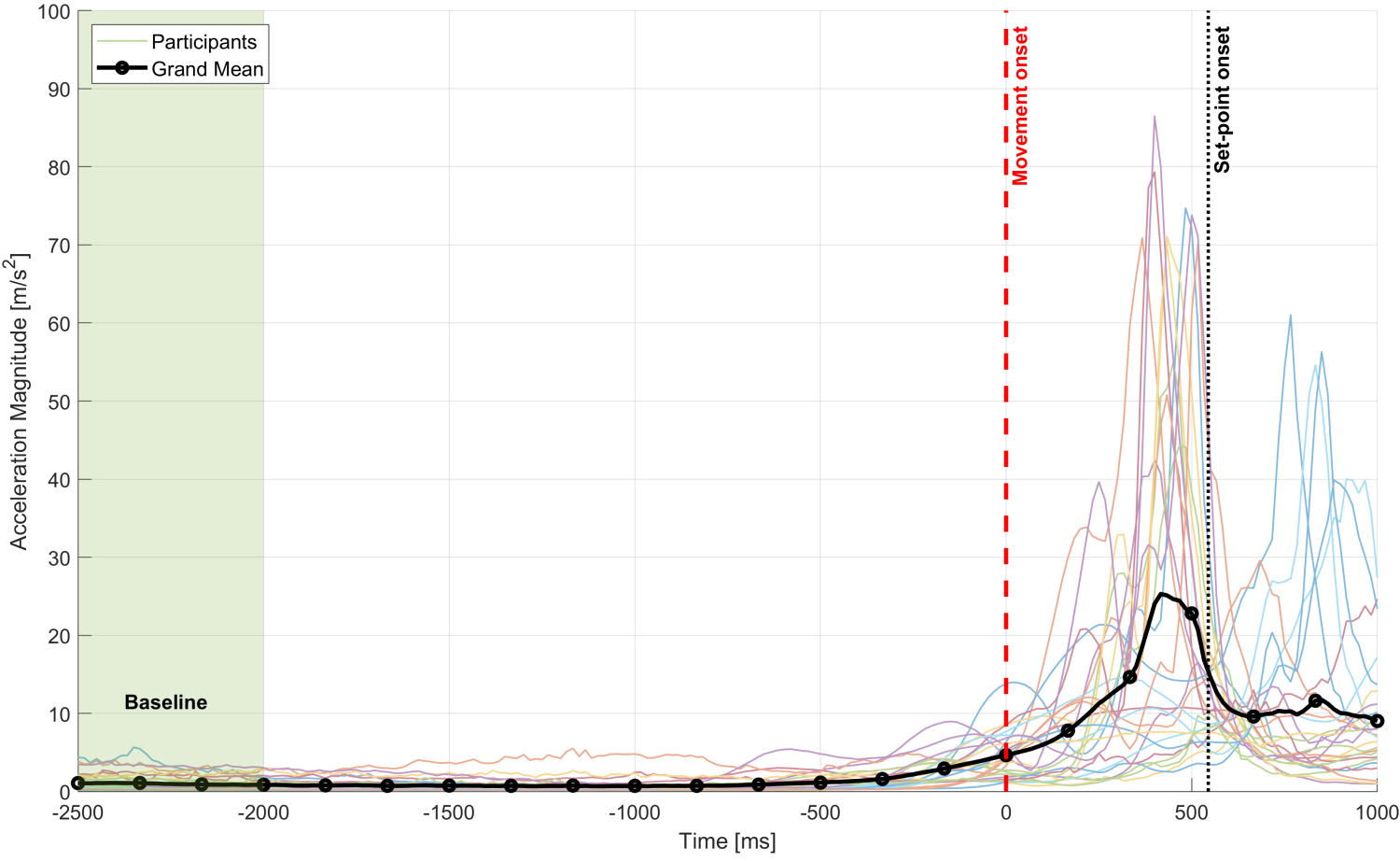

Supplement: Supplementary file 1 — Supplementary Material 1 [file 221_2026_7342_MOESM1_ESM.zip › Supplementary Information/Supplementary_Fig2.png]

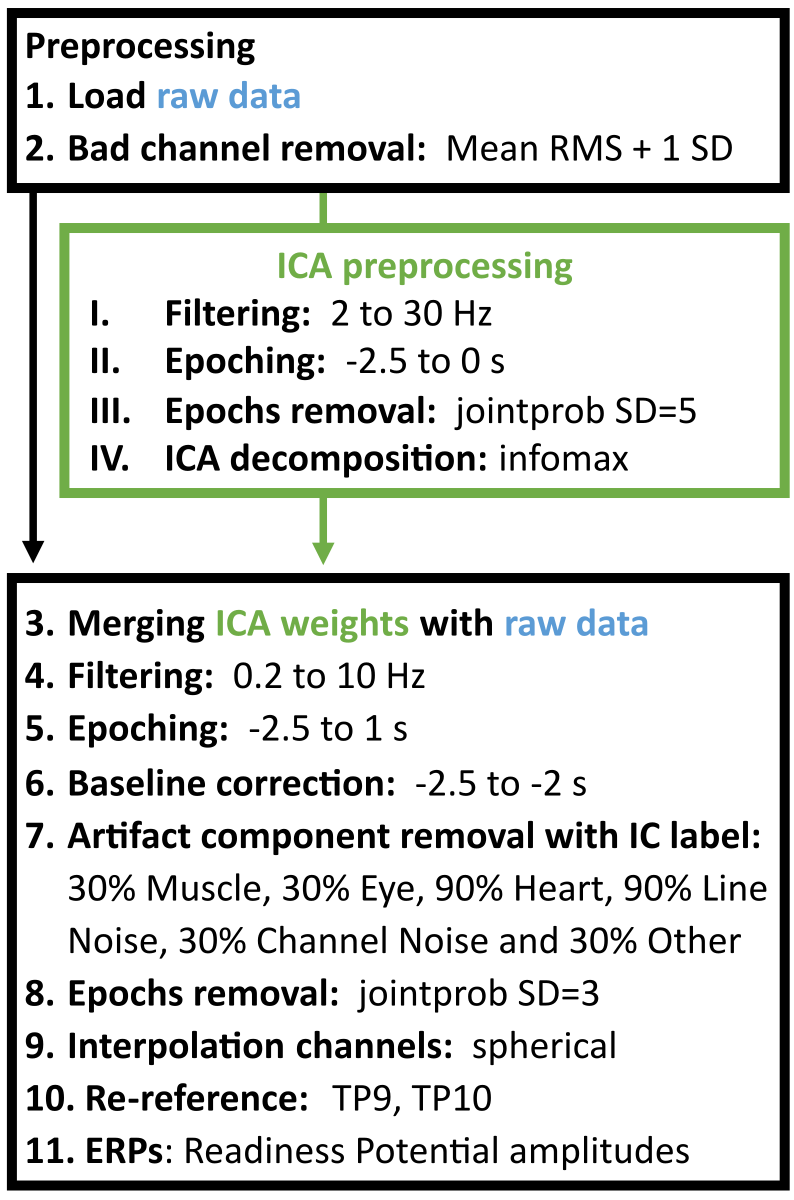

Supplement: Supplementary file 1 — Supplementary Material 1 [file 221_2026_7342_MOESM1_ESM.zip › Supplementary Information/Supplementary_Fig3.png]

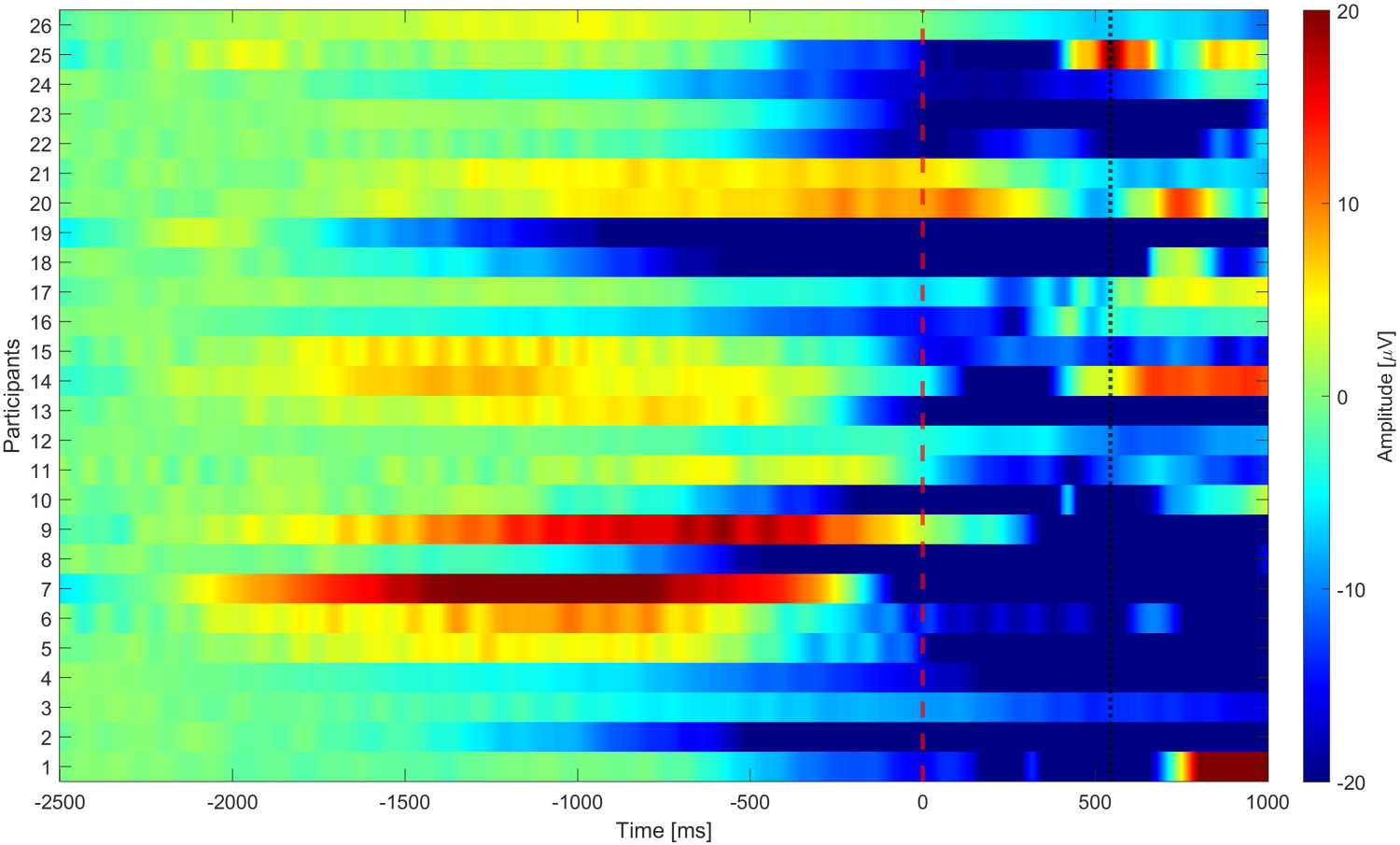

Supplement: Supplementary file 1 — Supplementary Material 1 [file 221_2026_7342_MOESM1_ESM.zip › Supplementary Information/Supplementary_Fig4.png]

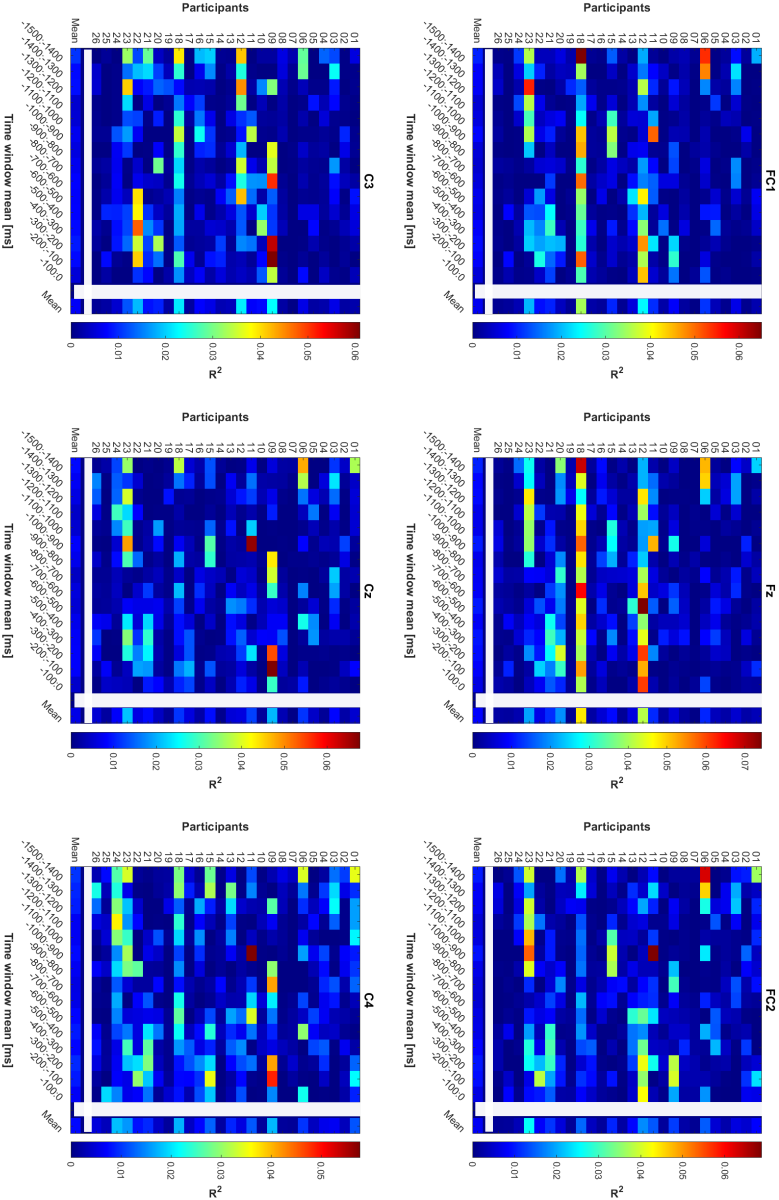

Supplement: Supplementary file 1 — Supplementary Material 1 [file 221_2026_7342_MOESM1_ESM.zip › Supplementary Information/Supplementary_Fig5.png]

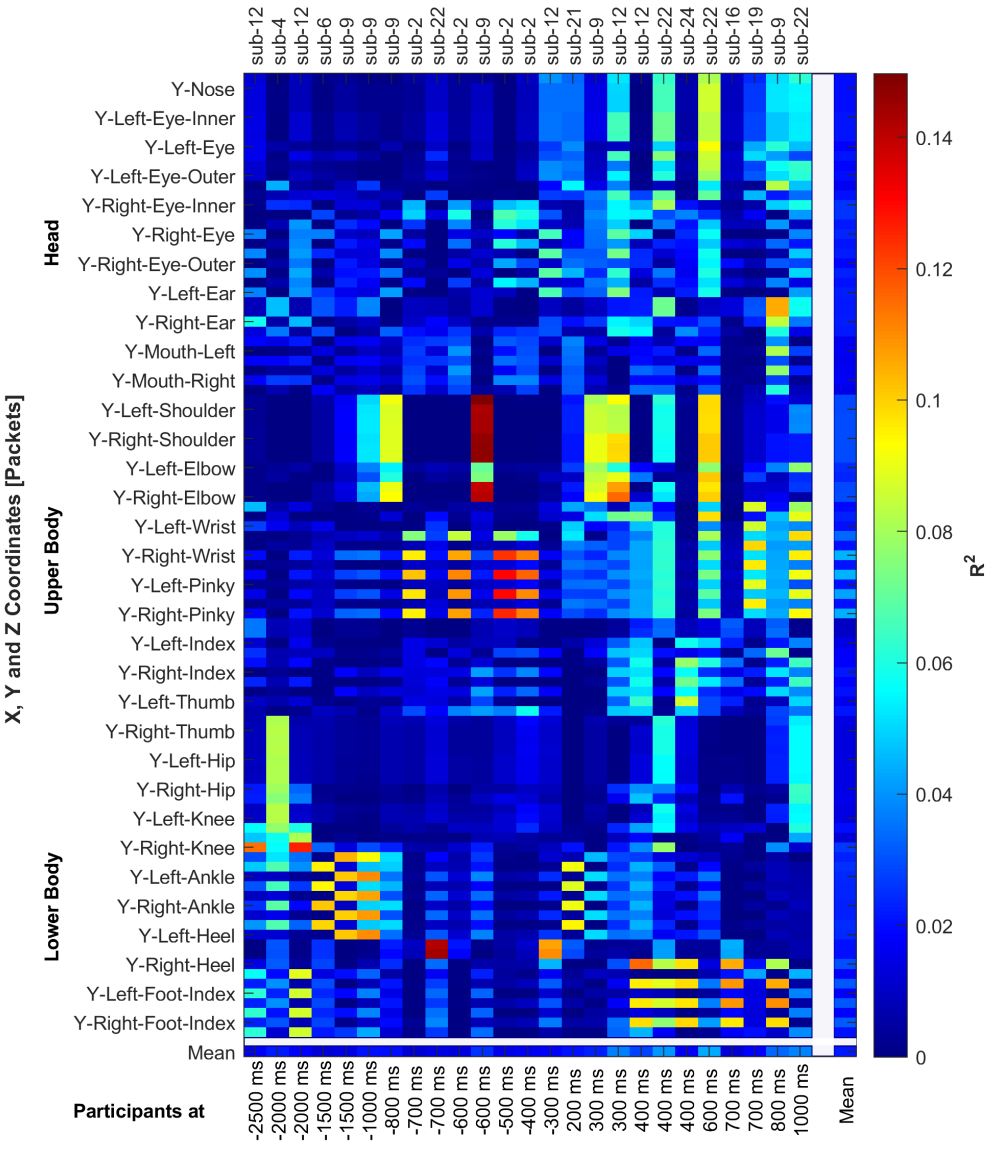

Supplement: Supplementary file 1 — Supplementary Material 1 [file 221_2026_7342_MOESM1_ESM.zip › Supplementary Information/Supplementary_Fig6.png]
